# Shared Biological Pathways between Antipsychotics and Omega-3 Fatty Acids: A Key Feature for Schizophrenia Preventive Treatment?

**DOI:** 10.3390/ijms22136881

**Published:** 2021-06-26

**Authors:** Ariel Frajerman, Linda Scoriels, Oussama Kebir, Boris Chaumette

**Affiliations:** 1 Institute of Psychiatry and Neuroscience of Paris (IPNP), Université de Paris, INSERM U1266, F-75014 Paris, France; ariel.frajerman@inserm.fr (A.F.); linda.scoriels@inserm.fr (L.S.); oussama.kebir@inserm.fr (O.K.); 2 GHU Paris Psychiatrie et Neurosciences, F-75674 Paris, France; 3 Department of Psychiatry, McGill University, Montréal, QC H3A 0G4, Canada

**Keywords:** antipsychotics, omega-3, membrane lipids, first episode psychosis, ultra high-risk patients, schizophrenia, oxidative stress, dopamine, glutamate, inflammation

## Abstract

Schizophrenia typically emerges during adolescence, with progression from an ultra-high risk state (UHR) to the first episode of psychosis (FEP) followed by a chronic phase. The detailed pathophysiology of schizophrenia and the factors leading to progression across these stages remain relatively unknown. The current treatment relies on antipsychotics, which are effective for FEP and chronic schizophrenia but ineffective for UHR patients. Antipsychotics modulate dopaminergic and glutamatergic neurotransmission, inflammation, oxidative stress, and membrane lipids pathways. Many of these biological pathways intercommunicate and play a role in schizophrenia pathophysiology. In this context, research of preventive treatment in early stages has explored the antipsychotic effects of omega-3 supplementation in UHR and FEP patients. This review summarizes the action of omega-3 in various biological systems involved in schizophrenia. Similar to antipsychotics, omega-3 supplementation reduces inflammation and oxidative stress, improves myelination, modifies the properties of cell membranes, and influences dopamine and glutamate pathways. Omega-3 supplementation also modulates one-carbon metabolism, the endocannabinoid system, and appears to present neuroprotective properties. Omega-3 has little side effects compared to antipsychotics and may be safely prescribed for UHR patients and as an add-on for FEP patients. This could to lead to more efficacious individualised treatments, thus contributing to precision medicine in psychiatry.

## 1. Introduction

Schizophrenia is a severe psychiatric disorder affecting more than 20 million individuals worldwide [1]. According to the well-established clinical staging model, schizophrenia is a progressive illness that typically emerges during late adolescence and transitions through several evolving stages: early vulnerability, at-risk mental state (also called ultra-high risk, abbreviated UHR), first episode psychosis (FEP), and chronic schizophrenia. The transition from one stage to the other is not inevitable, and it has been observed that only one-third of UHR individuals convert to psychosis after a 3-year follow-up [2]. The factors leading to progression across these stages remain largely unknown, reflecting the need to uncover the mechanisms underlying the pathophysiology of schizophrenia. The aetiology of schizophrenia is not restricted to brain dysfunctions, and the disorder is currently conceptualized as a systemic disease that includes immune, cardiometabolic, and endocrine abnormalities [3]. In addition to dopaminergic and glutamatergic abnormalities [4], patients with schizophrenia also experience increased levels of oxidative stress [5], inflammation, and immune reaction [6] and have abnormalities in membrane lipid composition [7] and in one-carbon (C1) metabolism [8].

The primary treatment for patients with schizophrenia relies on antipsychotics, which target almost exclusively the positive symptoms of the disorder, such as hallucinations, delusions, and paranoia. In the middle of the 20th century, chlorpromazine was discovered and used as the first antipsychotic treatment. The molecule acts as an antagonist of the dopaminergic receptors, and most of the first generation (typical) antipsychotics share this property [9]. Second-generation (atypical) antipsychotics have a broader molecular action compared to first-generation antipsychotics and target the dopaminergic, serotonergic, and (nor)adrenergic receptors [10]. These drugs also play a role in numerous other pathways [11,12] and, as a result, may lead to important side effects, including cardiac and metabolic dysfunction, weight gain [13], hyperprolactinemia [14] or extrapyramidal symptoms that may be associated with cognitive deficits [15]. Moreover, antipsychotics are relatively inefficient in the early phases of schizophrenia. Indeed, a recent trial with individuals at clinical high risk of psychosis showed that antipsychotics were associated with a higher conversion rate (26.9% vs. 17.7%, *p* = 0.035) [16]. The balance between risk and benefit in the use of antipsychotics suggests that it is a poor strategy to prevent the emergence of psychosis in vulnerable individuals. Preventive treatment should be prioritized in UHR individuals, considering that only one-third will develop psychosis. It is unacceptable that the remaining UHR individuals, who will not have a transition into the illness, should be exposed to the cumbersome side-effects of antipsychotics. Scientific effort has been made to develop new drugs with a better tolerance profile that could be used in preventive strategies. Nevertheless, illness stage-specific therapeutic strategies that prevent or delay the onset of this severely disabling disorder remain to be discovered.

**Figure 1:**
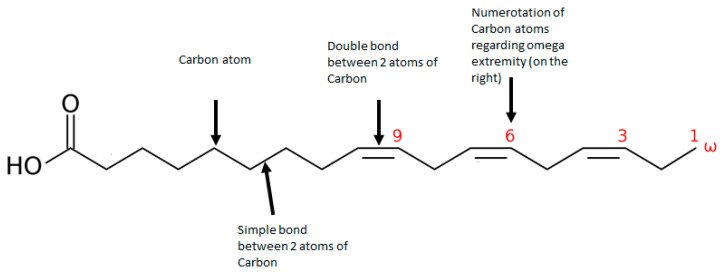
Representation of an omega 3 polyunsaturated fatty acid: α-linoleic acid (ALA).

Omega-3 fatty acids (omega-3) play a central role in brain functioning and may be a promising therapeutical alternative for vulnerable individuals. Omega-3 is an unsaturated fatty acid composed of a carboxylic acid with a long hydrophobic aliphatic chain, which has a first double bond on its third carbon (counting from the ‘CH3-end’ of the chain–Figure 1) [17,18]. Omega-3 belongs to monounsaturated (one bond) or polyunsaturated (up to six bonds) fatty acids (PUFA). α-linolenic acid (ALA) is an omega-3 PUFA that originates mainly from the diet and leads to the synthesis of other omega-3 PUFAs-through a series of metabolic cascades-including eicosapentaenoic acid (EPA) and the docosahexaenoic acid (DHA). However, their yields are relatively low, and supplements of EPA and DHA have been made available, mostly from fish oil extracts, to overcome potential deficits or an imbalance in omega-6, of which overconsumption has been associated with increased risk of inflammation and cardiovascular diseases [19]. In psychiatric disorders, a decrease in omega-3 levels has been uncovered in the neuron membrane of individuals with mood disorders and schizophrenia [20]. Research on lipid composition in cell membranes and in the serum of patients with schizophrenia has shown that higher levels of omega-3 are correlated with lower negative symptom severity, and with higher scores in cognition [21], although mixed results are found in the literature [22]. In UHR patients, lower levels of omega-3 and omega-3/omega-6 ratios (healthy ratio varies between 1-to-1 and 1-to-4) in erythrocyte membranes have been correlated with increased severity of depressive, psychotic, and general psychopathology symptoms and with increased cognitive impairment [23,24]. Thus, omega-3 supplementation has been proposed as a potential preventive treatment for UHR individuals as it might prevent transition to psychosis [25], and the supplementation appears to be safe and well tolerated [26].

In this review, first, we describe the role of omega-3 in different biological mechanisms involved in schizophrenia. We present how animal and in vitro studies have brought to light the comprehension of biological pathways and the action of antipsychotics and omega-3 in many different mechanisms and molecular cascades, showing their importance in the complex pathophysiology of schizophrenia. Second, we discuss the biological pathways omega-3 may share with antipsychotics in these biological systems. Last, but not least, we present the results of clinical trials on the effects of omega-3 supplementation in individuals with early and chronic schizophrenia. 

## 2. The Role of Omega-3 in the Plasma Membrane Composition and Dynamics 

The plasma membrane is a complex and dynamic structure, with a specific lipid composition and numerous proteins (including receptors) embedded within and on its surface. In this membrane, sphingolipids dynamically assemble with cholesterol to form lipids rafts that can include or exclude receptors selectively and confer an important role in cell communication [27,28,29,30]. Indeed, neurotransmitter activity is modulated by configuration and composition changes occurring in lipid rafts [31], of which the dynamics highly depend on PUFA composition [32,33]. It has been shown that this composition affects ligand binding properties, accelerates receptor endocytosis, and reduces open channel probability [31].

One of the hypotheses of the aetiology of schizophrenia lies on an imbalance in phospholipids and fatty acids (in particular PUFAs) in the membrane composition [34]. These abnormalities may be responsible for the hypo- and hyperactivation of several neurotransmitter systems such as the glutamatergic (N-methyl-D-Aspartate (NMDA) and α-amino-3-hydroxy-5-methyl-4-isoxazolepropionic acid (AMPA) receptors) and dopaminergic systems (D1 and D2 receptors). For instance, a study in vitro revealed a marked propensity of DHA to enhance the kinetics of oligomerisation of D2 and adenosine A2A receptors, which is important in dopaminergic activity in the striatum and has been shown to be decreased in schizophrenia [35].

Antipsychotics act upon membrane lipids at different levels. Modelling studies have shown that the insertion of antipsychotics into the lipid bilayer increases fluidisation and disorganises lipids of the plasma membrane [36]. In schizophrenia patients, a 3-month treatment with antipsychotics (mostly phenothiazine) showed that PUFA composition and omega-3/omega-6 ratios in erythrocyte membranes became comparable to those in healthy controls [37]. On the other hand, antipsychotics also appear to destabilise the activity of enzymes associated with lipid metabolism. For instance, it has been shown that chlorpromazine upregulates phospholipid transporters in human cells in vitro, leading to a loss in tight junctions and membrane integrity [38]. In rats, clozapine has been shown to disrupt sphingolipid homeostasis by decreasing hepatic ceramide and sphingomyelin levels. These effects were associated with hyperglycemia and hepatic glycogen reduction and may be responsible for the diabetogenic effect of clozapine [39]. Another study in rats has shown that risperidone up-regulates the expression of PUFAs (omega-3 and omega-6) in the membrane composition. Furthermore, all antipsychotics have been associated with an increase in membrane DHA (omega-3) levels and all antipsychotics, with the exception of quetiapine, have been associated with an increase in membrane arachidonic acid (omega-6) levels [40]. In patients with schizophrenia, haloperidol has been shown to increase PUFA biosynthesis and total incorporation of arachidonic acid in platelets [41]. 

The insertion of omega-3 in the plasma membrane can modulate lipid–lipid and lipid–protein interactions [42] and leads to modifications in membrane properties and receptors activity. For instance, membrane modelling studies have shown that the substitution of an omega-9 fatty acid (oleic acid) by an omega-3 fatty acid (DHA) decreases phospholipase A2 (PLA2) activity [43]. This enzyme cleaves PUFAs that are stored in the membrane phospholipids [44] and plays important roles in several biological mechanisms [45], including membrane lipid functioning and inflammation [46], which lead to modifications in membrane elasticity, lipid organization, and vesicle formation [43]. PLA2 appears to be hyperactivated in patients with schizophrenia [47] and this effect can be reduced by antipsychotics [48]. Similarly, omega-3 supplementation also appears to decrease PLA2 activity in UHR patients [49]. 

More generally, EPA supplementation in patients with chronic schizophrenia has revealed a decrease in saturated fatty acids and mono unsaturated fatty acids levels and an increase in omega-3 and omega-6 levels in the plasma membrane of patients’ erythrocytes [50]. In UHR patients, EPA and DHA supplementation also promotes the incorporation of omega-3 in the erythrocyte plasma membrane, at the expense of omega-6 [51].

As previously mentioned, the changes induced by omega-3 in the plasma membrane are directly implicated in the modulation of membrane receptors and in particular, neurotransmitters receptors. The following three chapters discuss in further detail the effects of omega-3 on the endocannabinoids, dopaminergic, serotonergic, and glutamatergic neurotransmission systems. 

## 3. Effects of Omega-3 on the Endocannabinoid System

Endocannabinoids are endogenous lipid-based neurotransmitters that bind to cannabinoid receptors (CB1 and CB2). They can modify the membrane biophysics, notably its fluidity [52], and modulate oxidative stress and lipid peroxidation, via their action on CB1 and CB2, and also on peroxisome proliferator-activated receptor alpha (PPARα-a key regulator of lipid metabolism) [53]. The endocannabinoid system has also been associated with modulators of inflammation, such as interleukin 6 (IL6) and kynurenine metabolites [54]. Endocannabinoids are directly synthesised from a variety of membrane fatty acids, in particular arachidonic acid, which yields two main independent arachidonic acid derivatives: anandamide and 2-arachidonoyl-sn-glycerol (2AG). These two molecules are involved in brain neuromodulation, and their deregulation has been associated with schizophrenia [55]. 

Studies in individuals with FEP and chronic schizophrenia have identified an elevation of anandamide and 2AG concentrations in their cerebrospinal fluid [56,57]. This has been confirmed in a recent meta-analysis that examined endocannabinoid system metabolites in blood and cerebrospinal fluid in patients with schizophrenia [55]. However, a post-mortem study found decreases of anandamide in the cerebellum, hippocampus, and prefrontal cortex of patients with schizophrenia [58]. This apparent divergence reveals the complexity of this biological system, which further depends on comorbidity factors, such as depression status or drug abuse. More recently, studies have suggested that endocannabinoids may also be involved in the hypoactivation of the NMDA receptor associated with schizophrenia [59]. 

Cannabidiol (CBD) is an exogenous cannabinoid that has direct and indirect antioxidative effects [60,61] and targets a variety of receptors [62,63], including CB1 and CB2 receptors [64,65]. Although CBD has a low affinity for these receptors, it appears to act as a negative allosteric modulator of CB1 and a partial agonist of CB2 receptors [66,67,68]. CBD has been tested for its antipsychotic properties in three studies, one of which revealed an activity similar to amisulpride (a D2 and D3 receptor antagonist antipsychotic) and two that showed no effect on efficacy or cognition in individuals with schizophrenia [69]. Both CBD and omega-3 appear to modulate the PPARα signalling pathway that regulates mesocorticolimbic dopamine activity and counteracts neuropsychiatric symptoms [70].

In mouse model studies, it has been observed that omega-3 deficiency impairs the endocannabinoid system, notably with a reduction of the presynaptic CB1 receptor function in the prefrontal cortex and the nucleus accumbens [71,72]. These changes lead to reduced social interactions and increased immobility, revealing typical symptoms of anxiety and depression [71,72]. Further, a diet deficient in omega-3 induces an increase in the levels of anandamide and 2AG in the brain [73], which is compensated by omega-3 supplementation 

As described above, the role of omega-3 in the cannabinoid neurotransmission system is of importance, although it may not be the most relevant interaction with regards to schizophrenia, of which the molecular hypothesis lies mainly in the hyperactivation of the dopaminergic system in the limbic areas and the hypoactivation of the dopaminergic system in the cortical areas of individuals with schizophrenia.

## 4. Effects of Omega-3 on the Dopaminergic and Serotonergic Systems

The dopaminergic system is a network of neurons containing dopamine receptors that are involved in several cognitive mechanisms, including learning, executive functions, and motivation. One of the most important and characterised biological theories in schizophrenia lies in the hyperactivation of the dopaminergic neurons in the mesolimbic areas of the brain, leading to the positive symptoms of schizophrenia, and in the hypoactivation of the dopaminergic neurons in the frontal areas of the brain, leading to the negative and cognitive symptoms of schizophrenia [74]. 

Chlorpromazine, the first antipsychotic discovered to treat individuals with schizophrenia, targets dopaminergic neurons and is efficient at reducing positive symptoms, such as delirium, paranoia, and hallucinations. After chlorpromazine, several antipsychotics were discovered and some of them target the dopaminergic system, although many other neurotransmitter systems are also involved [75]. First-generation antipsychotics are antagonists of D2 receptors, whereas second-generation antipsychotics reduce both dopaminergic and serotonergic neurotransmitters and convey better treatment efficacy [75]. Clozapine, olanzapine, and quetiapine exert a modest action upon D2 receptors, whereas risperidone, paliperidone, sertindole, and lurasidone exert a potent action on these receptors [75].

Research over the last two decades has demonstrated that the variation in omega-3 levels is positively correlated with dopamine concentration and the number of D2 receptors in the brain (but not D1) [76]. A study in healthy humans and patients with alcohol abuse has shown that levels of omega-3 in the cerebrospinal fluid are positively correlated with concentrations of dopamine and serotonin metabolites [77]. Another study in individuals suffering from major depression also established positive correlations between plasma omega-3 and molecules from the dopaminergic system, but not those from the serotonergic system [78]. There are no studies, to our knowledge, that have analysed the effects of omega-3 on the dopaminergic system in patients with schizophrenia. In an Alzheimer’s disease rat model, omega-3 deficiency led to abnormalities in the dopamine metabolism and in behaviour, leading to increased anxiety, hyperactivity, reduced behavioural flexibility, and memory impairment [79,80,81]. Brain rat studies in an omega-3 deficiency diet also showed changes in dopamine internalization in the frontal cortex [82] and a reduction in central serotonin synthesis [83]. The deficiency diet appeared to cause hypoactivation of dopamine in mesocortical pathways, with a decrease in mRNA expression of D2 receptors in the frontal cortex, and a hyperactivation of dopamine in mesolimbic pathways, with an increase in mRNA expression of D2 receptors in the nucleus accumbens [84]. This dopaminergic activity pattern is similar to what is described in the aetiology of schizophrenia, which could potentially lead to the discovery of new animal models for the dopaminergic theory of schizophrenia. 

The imbalance created with omega-3 depletion can be inverted using omega-3 supplementation [85]. For instance, omega-3 supplementation prevents molecular changes caused by amphetamine, one of the few pharmacological animal models for the dopaminergic theory of schizophrenia. Amphetamine induces dopaminergic deficits and increases the levels of D1 and D2 receptors, which can be inverted by omega-3 supplementation. Indeed, omega-3 induces a reduction in D1 and D2 receptors levels and an increase in the levels of vesicular monoamine transporter 2, which transfers dopamine from the cytosol to synaptic vesicles in the prefrontal cortex (PFC) [86]. Conversely, an excess of saturated fatty acids, from childhood to adulthood, seems to increase sensibility to amphetamine by increasing the bursting activity of dopaminergic neurons in the mesolimbic pathway [87]. The effects of omega-3 supplementation on the dopaminergic system have also been studied in a Parkinson’s animal model and show that omega-3 increases striatal dopamine synthesis and specifically reverses dopamine loss in the nigrostriatal pathway, with no effect in other–mesolimbic or mesocortical–pathways [88].

As described above, omega-3 acts upon the dopaminergic system and it has been established that this neurotransmission system has multiple interactions with the glutamatergic system, which are potentially relevant therapeutic targets for schizophrenia [89].

## 5. Effects of Omega-3 on the Glutamatergic System

The glutamatergic system is the most prevalent excitatory system in the brain. It plays a role in information processing in neuronal networks and is involved in several cognitive functions. It has been observed that lipid rafts are important for the activity of NMDA receptors, which are one of the ionotropic glutamate receptors [90,91]. Membrane cholesterol appears to have an effect on the structural dynamics of the metabotropic glutamate receptor (mGluR1) [92], and reciprocally, glutamate receptors modulate brain membrane lipid composition. Indeed, in vitro and in vivo studies have shown that a short stimulation of the glutamatergic neurotransmission system induced a significant loss in membrane cholesterol [93]. In vitro, blockers of NMDA receptor (MK801) and mGluR5 (MPEP) appear to attenuate the glutamate-induced loss of cholesterol and elevation of 24S-hydroxycholesterol.

In schizophrenia, there is a theory on the hypofunction of the glutamatergic system that complements the dopaminergic theory discussed above. Indeed, the disorder is characterised by a reduction in glutamate metabolites that explain some of the negative and cognitive symptoms present in the early and chronic stages of the disorder [94,95].

Antipsychotics target positive symptoms in individuals with schizophrenia and it is still unclear how they act on other symptoms. A systematic review has reported that antipsychotic use is associated with lower levels of glutamate in patients with schizophrenia [96]. 

The interaction between PUFAs and the glutamate NMDA receptor has been established for decades [97]. Research has demonstrated that omega-3 deficiency affects the maturation of glutamatergic synapses in rats, which leads to memory deficits and anxiety-like behaviour [98]. It also appears to aggravate age-induced degradation of glutamatergic transmission in rat hippocampal CA1 [99], probably by modulating NMDA activity [100] and reducing brain derived neurotrophic factor (BDNF) levels, which are involved in memory and learning [101]. A study on a transgenic mouse model of Alzheimer’s disease has shown that omega-3 deficiency decreases the subunits of NMDA receptors NR2A and NR2B [102]. These results were also found in vitro, in addition to an inhibition of neurite growth and synaptogenesis [103] and a reduction in several glutamate receptor subunit concentrations (GluA1, GluA2, and NR2B), and in synaptic vesicle proteins in hippocampal synaptosomes [104]. There are several schizophrenia animal models based on glutamatergic dysfunction that use non-competitive NMDA receptor antagonists (e.g., dizocilpine (MK 801), phencyclidine (PCP) or ketamine) [105]. These drugs induce impairments in several cognitive and social functions, including attentional, memory, and executive function impairments and deficits in social interaction [105,106]. A schizophrenia mouse model has recently shown that NMDA receptor hypofunction affects brain lipid composition and induces increases in omega-6, possibly via PLA2 activation. The cumulative effects of NMDA receptor hypofunction and an omega-3 deficient diet led to an increase in mortality [107]. A mouse model mimicking omega-3 deficiency has also shown damage in NMDA mGluR5 receptors, which consequently impair mGluR5-endocannabinoid-mediated synaptic plasticity and have deleterious effects on cognitive and emotional functions [108]. 

In vitro studies from a variety of neuropsychiatric disorders models show that the addition of omega-3 generally prevents potential glutamatergic system deleterious effects. For instance, the addition of DHA in hippocampal slices from an Alzheimer’s disease rat model has shown a reduction in glutamate AMPA receptor-mediated cell death [109] and a decreased activity of astroglial glutamate transporters (GLAST and GLT-1). In rat astrocytes, DHA reduces glutamate uptake [110]. 

Omega-3 supplementation in NMDA hypofunction rat models has been associated with reduced excitotoxic brain damage induced by NMDA antagonists [111,112]. Similar results were obtained with the administration of hydroxy docosahexaenoic (hDHA–a DHA derivative) in a transgenic mouse model of Alzheimer’s disease [113]. This was also supported by studies based on rat models of schizophrenia using ketamine, in which omega-3 prevented damages caused by the drug, notably damages in the schizophrenia-like positive, negative, and cognitive symptoms [114,115]. 

To summarise, many studies—mostly in animals—describe associations between omega-3 and the cannabinoids, dopaminergic, serotonergic, and glutamatergic neurotransmission systems. They show that the levels of omega-3 affect these brain neurotransmission systems and that a combined effect of omega-3 deficiency with genetic or environmental factors may increase the risk of developing neuropsychiatric disorders such as schizophrenia or Parkinson’s disease [74]. The efficacy of these neurotransmission systems depends on the well-functioning network between neurons, which is partly performed by myelin.

## 6. The Role of Omega-3 in Myelin Constitution and Function

Myelin is a lipid-rich substance produced by Schwann cells in the peripheral nervous system (PNS) and oligodendrocytes in the central nervous system (CNS). Myelin is located in the white matter and constitutes a fatty sheath around nerve cell axons and allows electrical impulses to transmit rapidly and efficiently along the nerve cells. 

The pathophysiology of schizophrenia involves alterations in white matter, and in particular in myelination. Indeed, myelin appears to be significantly reduced in post-mortem schizophrenia and in vivo brain imaging studies [116]. Research on post-mortem brains found an increase in sphingolipids and phosphatidylcholine plasmalogens, molecules that are implicated in myelination, in the frontal cortex of patients suffering from schizophrenia compared with healthy controls or patients suffering from amyotrophic lateral sclerosis [117]. In schizophrenia patients, membrane erythrocyte PUFA levels are significatively correlated with myelin integrity. A study on 30 patients with early onset of schizophrenia found positive correlations between total membrane erythrocyte PUFA levels and fractional anisotropy, which is an imaging marker reflecting the integrity of the myelin. Lower levels of nervonic acid (omega-9) and fractional anisotropy values were associated with more negative symptoms [118].

An imbalance in sphingolipids, and in particular in sphingomyelin, leads to a decrease in cerebral myelination. As a consequence, neuronal membranes properties are also potentially altered and may jeopardise the efficiency of the electrical signal transmission [119]. Acting on myelin dysfunction and its associations with NMDA receptors and oxidative stress is one of the therapeutic hypotheses currently studied for the treatment of schizophrenia [120]. 

In a rat model of schizophrenia, hypomyelination has been shown to affect parvalbumin interneurons and to lead to cognitive symptoms [121]. In mice, the induction of demyelination by cuprizone (copper chelator) leads to motor and cognitive disorders, similar to those observed in schizophrenia, affecting working memory, pre-pulse inhibition (PPI), and social interactions. These effects are age-dependent: exposure at early age causes memory deficits immediately and after remyelination, while late exposure results in immediate deficits only [122]. These results suggest that myelin damage has lasting consequences if it occurs during childhood or early adolescence, supporting the neurodevelopmental aetiology theory of schizophrenia. 

Quetiapine, an atypical antipsychotic, is able to improve cuprizone-induced cognitive impairment and partly normalise cuprizone-induced lipid changes in mice [123]. In rats, sphingolipid metabolism that is jeopardised in schizophrenia can be altered by antipsychotics [39,124]. In patients with schizophrenia, it appears that the choice of antipsychotic treatment may differentially impact brain myelination [125,126]. An imaging study has shown that risperidone (a second generation antipsychotic) is associated with higher white matter volumes, a proxy for myelination, compared to fluphenazine (a first generation antipsychotic) [125]. 

In animals, deficiencies in omega-3 or cholesterol [127] lead to myelinisation abnormalities and impact neuronal activity [128]. EPA (omega-3) supplementation is able to protect against demyelination and brain growth damage in young (21–35 post-natal days) rats artificially exposed to demyelination by cuprizone feeding [129]. 

The role of omega-3 in the different biological mechanisms described above mainly involves neuronal pathways. Additionally, omega-3 also appears to play an important role in other biological mechanisms, e.g., inflammation and oxidation pathways, that have been identified as dysfunctional in schizophrenia.

## 7. The Effect of Omega-3 in Anti-Inflammatory and Antioxidant Pathways

Lipids, via fatty acids or other mediators, have an important and complex role in inflammation. Arachidonic acid (omega-6) derivatives, called eicosanoids (for example: prostaglandins or thromboxanes) have pro-inflammatory effects. DHA (omega-3) derivatives, called docosanoids (for example: resolvins or protectins), have anti-inflammatory and antioxidant properties [130]. In vitro data show an inhibitory action of omega-3 against T-cell proliferation and a decrease in interleukin 2 (IL_2_), a pro-inflammatory cytokine playing a role in T-cell differentiation [131,132]. Inflammation can increase concentrations of reactive oxygen species (ROS) and, oxidative stress can, in turn, increase inflammation [133].

Individuals suffering from schizophrenia have increased levels of inflammation [134] and oxidative stress [5,135] that are present from early stages of the disorder (FEP) [136]. Research has shown that plasma total antioxidant status is inversely correlated with cognitive impairment in both patients with chronic schizophrenia and FEP [137,138]. Antipsychotic actions in inflammation are complex but they generally appear to reduce levels of pro-inflammatory cytokines [139]. Antipsychotics also appear to reduce oxidative stress [140].

In rodents, it has been shown that omega-3 decreases inflammation and omega-6 increases it. Thus, an omega-3 deficiency leads to a higher vulnerability to inflammation, while an increase in the omega-3/omega-6 ratio has a protective effect [141,142]. These effects appear to involve the IL_6_ signalling pathway [143]. Recently, research on rats found that omega-3 supplementation reduced inflammation (via a decrease in the expression of toll-like receptor 4, NF-κB, TNFα, IL_1b_, and IL_6_) and oxidative stress (via an increase in superoxide dismutase and glutathione (GSH) activity and a decrease in malondialdehyde, oxidized glutathione (GSSG), and the GSSG/GSH ratio) induced by lipopolysaccharides in neonatal rat brain, whereas omega-6 had the opposite effect [144]. In a rat model of ketamine-induced schizophrenia, supplementation of omega-3 (DHA + EPA) prevented an increase in acetylcholinesterase activity [145] and lipid and protein degradation associated with oxidative stress [115]. 

In healthy active and sedentary individuals, omega-3 supplementation significantly decreases malondialdehyde (MDA–an oxidative stress marker) and TNFα (an inflammation cytokine involved in necrosis and apoptosis) [146]. Several meta-analyses have been performed over the past few years and they have reported that omega-3 supplementation significantly improves total antioxidant capacity, increases glutathione, and reduces MDA [147], and omega-3 and vitamin E co-supplementation also increases total antioxidant capacity and nitric oxide and reduces MDA levels, but has no impact on super oxide dismutase and catalase activity or on glutathione concentrations [148]. In bipolar patients, it has been reported that their plasma IL_6_ and TNFα levels are inversely correlated with EPA levels [149], and a study suggests that inflammation may predict the response to omega-3 supplementation in depression [150]. In schizophrenia patients, adding anti-inflammatory therapy to antipsychotic could be useful [151,152]. In FEP, omega-3 supplementation increases the total antioxidant capacity [153]. In UHR patients, omega-3 supplementation alters the rate of the circulating soluble form of the intercellular adhesion molecule one (sICAM-1), IL_6_, and sIL_2_R [154]. In a study on UHR patients, it has been reported that omega-3 supplementation significantly increases alpha-tocopherol and decreases total glutathione [155]. This action may be partially due to the effect of omega-3 supplementation on the activity of PLA_2_ [49].

Last, but not least, omega-3 appears to also play a role in one-carbon metabolism, a biological pathway that is connected to several other biological mechanisms and appears to be involved in the pathophysiology of schizophrenia.

## 8. Effect of Omega-3 on the One-Carbon Metabolism 

One-carbon metabolism includes molecules from the methionine and folate cycles (for example: vitamin B12, folic acid and homocysteine) and is involved in many biological functions such as oxidative stress, DNA methylation, histone modification, biosynthesis of phospholipids, among others [156,157]. Animal studies have demonstrated that a folate/methyl-deficient diet results in perturbations in the levels of one-carbon metabolites, leading to oxidative stress and oxidative DNA damage in the brain [158]. In an animal study, vitamin B-deprived rats had a decrease in omega-6 and omega-3, as well as a decreased ability to convert phosphatidylethalonamine into phosphatidylcholine [159] and in the transport of brain DHA [160]. The role of homocysteine in brain membrane lipid composition has also been confirmed in chicken, and exposure to excessive homocysteine results in a decrease in their brain mass [161]. In healthy humans, it has been shown that plasmatic homocysteine levels are negatively correlated with plasma phospholipid concentration in EPA, DHA, total omega-3, and the omega-3/omega-6 ratio and are positively correlated with arachidonic acid [162], thus confirming the protective effects of omega-3.

In medication-naïve FEP patients, plasma levels of folate and vitamin B12 are lower and homocysteine higher than in healthy controls [163]. Risperidone decreases homocysteine levels in FEP [164]. 

Omega-3 plays a role in the pathways that involve one-carbon metabolism and oxidative stress [165]. Research on rats submitted to early stress (maternal deprivation) or late life stress (chronic mild stress) revealed that omega-3, N-acetylcysteine, and folic acid supplementation have antioxidant effects (decreased levels of protein carbonylation, lipid peroxidation, nitrite/nitrate concentration, and myeloperoxidase activity; increased superoxide dismutase and catalase activities) in rats brains under both stress conditions [166]. Further, omega-3 and folic acid supplementation can prevent depressive-like behaviour (increased immobility time during a forced swimming test) induced by late life stress in rats [166]. In pregnant rats, an imbalance in maternal micronutrients (excessive maternal folic acid supplementation on a B12 deficient diet) induced an increase in oxidative stress and a reduction in brain DHA levels in pups. These effects were counterbalanced by omega-3 supplementation [167]. A meta-analysis on omega-3 supplementation describes a decrease in homocysteine levels, which are potentialized by the addition of vitamin B (B6, B9, and B12) [168]. In patients with cognitive impairment and Alzheimer disease, omega-3 and vitamin co-supplementation have been shown to improve cognition [169,170]. 

## 9. Omega-3 Overall Actions and Shared Biological Pathways with Antipsychotics 

**Figure 2:**
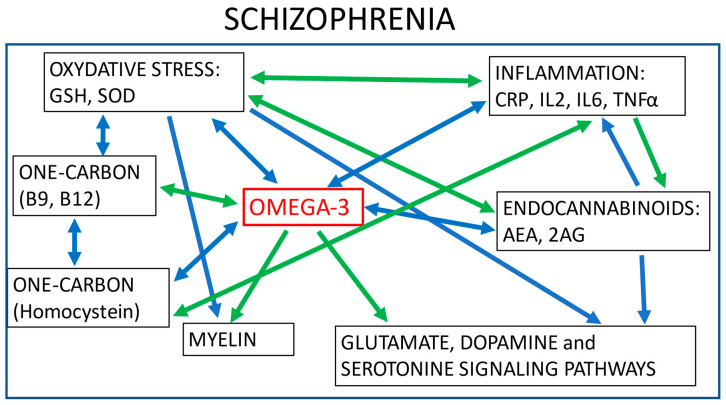
Interactions between different biological pathways involved in schizophrenia. Green arrows: positive correlations; blue arrows: negative correlations; GSH: glutathione; SOD: superoxide dismutase; CRP: C reactive protein; IL_2_: Interleukin 2; IL6: Interleukin 6; TNFα: Tumor Necrosis Factor; AEA: anandamide; 2AG: 2-arachidonoyl-sn-glycerol.

As discussed in the first part of this review, omega-3 is involved in several biological pathways and could be considered a gateway to the complex pathophysiology of schizophrenia, which includes oxidative stress, inflammation, myelination, glutamate and dopamine signalling pathways, one-carbon metabolism, and endocannabinoid pathways. These biological pathways also interact with each other, further increasing complexity. Indeed, it has been established that inflammation is associated with oxidative stress and endocannabinoids, oxidative stress is associated with one-carbon metabolism, and endocannabinoids are involved in glutamate and dopamine signalling pathways, for example (Figure 2). To add to this complexity, there may be a time window that potentiates the action of omega-3, as it may play a role against the emergence of psychosis through its neuroprotective effects, especially during adolescence, when brain maturation takes place [171].

Additionally, the actions of antipsychotics and omega-3 supplementation have been thoroughly described for each biological pathway considered and show that the two molecules have some shared properties. These actions are summarised in Table 1. Human studies have enabled us to correlate these data with clinical symptoms. Unlike antipsychotics, omega-3 supplementation has very few side effects, mainly related to minor gastrointestinal adverse events [26]. Individuals with schizophrenia have different patterns of response to antipsychotics, depending on the category of the antipsychotic and also on individual variability, which may be related to specific intermediate phenotypes. It is likely that the same conjectures apply to omega-3. Indeed, several studies in schizophrenia find evidence for two patient subgroups: one with membrane lipid abnormalities and one without [21,172,173]. In UHR patients, one study suggested the levels of alpha-linolenic acid (omega-3) are a potential biomarker for omega-3 supplementation response [174]. Technical progress is leading to a better understanding of lipids and to the possibility of using them for personalised medicine [175].

## 10. Omega-3 Supplementation at Different Stages of Schizophrenia

As previously described, omega-3 supplementation has been used in several trials for UHR, FEP, and chronic schizophrenia patients. Meta-analyses on the effect of omega-3 used as a supplement at different stages of schizophrenia suggest that the supplement is efficacious at reducing symptoms in the earlier stages of schizophrenia but results are mixed for chronic schizophrenia patients [179,180]. Indeed, some studies on chronic schizophrenia patients find that omega-3 supplementation improves symptomatology and prevents some of the antipsychotic side effects [50,181,182], while others find no efficacy of omega-3 supplementation in the stable or acute phases of the disorder [183,184]. In FEP, a clinical trial found that the efficacy of the supplementation was dependent on the dose and the length of the supplementation, with higher doses and longer periods of supplementation being associated with better clinical outcomes [185,186]. In UHR patients, omega-3 supplementation has been shown to reduce the risk of psychotic transition (27.5% vs. 4.9%; *p* = 0.007) [187] with a long-lasting effect (seven years follow-up) [188], although a more recent multi-center study shows a lack of effect of omega-3 supplementation [189]. However, this multi-center cohort presented lower rates of psychotic transition, which may have been attributed to several biases related to compliance with the treatment, medication confounders or the absence of a food questionnaire or assessment of differences in PUFA membrane levels [190]. Indeed, almost 60% of participants had not taken the omega-3 supplement as required, and a secondary analysis that compared compliant versus non-compliant participants showed that the cumulative psychotic conversion rate within one year was significantly higher in the non-compliant group compared to the compliant group (14.8% vs. 4.7%; *p* < 0.001) [191]. Actually, the research on this topic is relatively scarce and has brought some debate on the most appropriate source of omega-3 supplementation between DHA (22:5 PUFA) and EPA (20:5 PUFA) [192], and on the heterogeneity of patients, as several studies suggest that only a subgroup of patients have lipid abnormalities [172,173,193,194].

## 11. Conclusions

This review is a contribution to our understanding of the role played by omega-3 fatty acids in different biological pathways that are involved in schizophrenia. Exploring the biological pathways omega-3 is involved in improves our knowledge about the mechanisms underlying the pathophysiology of schizophrenia. We show that omega-3 supplementation is involved in mechanisms and has biological actions similar to antipsychotics. However, omega-3 does not present the cumbersome side effects that antipsychotics do. Further, omega-3 appears to be efficient as soon as the first symptoms of schizophrenia emerge, in UHR patients, a phase in which antipsychotics are the least effective. Thus, omega-3 supplementation has been proposed as an alternative to antipsychotics in this very early stage. In FEP patients, omega-3 appears to improve recovery and could be prescribed as add-on to their current medication. The low cost associated with omega-3 supplementation makes it a realistic and relatively easy treatment to implement. While omega-3 supplementation appears to be a promising therapeutic strategy, it is possible that only a subgroup of the individuals would benefit from this treatment. Further research is needed to determine the biological factors justifying this supplementation, paving the way to a more personalised medicine.

## Figures and Tables

**Table 1:** The biological effects of antipsychotics and omega-3 supplementation in patients with schizophrenia.

	Schizophrenia Patients with No Treatment	Effect of Antipsychotics	Effect of Omega-3 Supplementation
**Biological systems**			
Dopamine and glutamate neurotransmission pathways	Altered [4]	Rebalanced [96,176]	Rebalanced [88]
Myelination	↓ [116]	↑ [125]	↑ [129]
Inflammation	↑ [134]	↓ [139]	↓ [12]
Oxidative stress	↑ [135]	↓ [140]	↓ [177]
**Molecules**			
Homocysteine	↑ [163]	↓ [164]	↓ [168]
B9, B12	↓ [163]	-	Positive correlation [162]
Phospholipase A2	↑ [47]	↓ [48]	↓ [49]
Endocannabinoids (anandamide and 2AG)	↑ [62]	-	↓ [178]
